# Complete Genome Sequence of *Lily Symptomless Virus*, Isolated from *Alstroemeria* Plants in Ecuador

**DOI:** 10.1128/MRA.00368-19

**Published:** 2019-06-27

**Authors:** Francisco Flores, Patricia Garrido, Fiama Guevara, Roberto Granda

**Affiliations:** a Centro de Investigación de Alimentos (CIAL), Facultad de Ciencias de la Ingeniería e Industrias, Universidad UTE, Quito, Ecuador; b Departamento de Ciencias de la Vida y Agricultura, Universidad de las Fuerzas Armadas–ESPE, Sangolquí, Ecuador; c Grupo de Protección Ambiental, Facultad de Ciencias de la Ingeniería e Industrias, Universidad UTE, Quito, Ecuador; Portland State University

## Abstract

In this work, we present the complete genome sequence of Lily symptomless virus isolated from Alstroemeria plants. The genome is 8,391-bp long, arranged into six open reading frames (ORFs), with a 5′-untranslated region (5′-UTR) of 55 nucleotides (nt) and a 3′-UTR of 48 nt.

## ANNOUNCEMENT

The most important cut flowers worldwide are roses and chrysanthemums, followed by various bulbs, with Tulipa and Lilium spp. being the most valuable (1). Ecuador is the world’s third largest exporter of cut flowers, 26% of which are summer flowers. The volume of exported lilies in 2017 was equivalent to over 1,000 metric tons (2). Over 11 viral diseases have been reported to infect lilies (3). Lily symptomless virus (LSV), a member of the *Carlavirus* genus of the Betaflexiviridae family, is one of the most common viruses in lilies and has spread worldwide with the movement of infected plant material (4).

In November 2018, mosaic symptoms were observed in cultivated Alstroemeria (Peruvian lily) plants in Lasso, Province of Cotopaxi, Ecuador. One hundred milligrams of infected leaf samples was collected from symptomatic plants, and total RNA was extracted using the PureLink RNA minikit (Invitrogen), according to the manufacturer’s instructions. Libraries were prepared from total RNA using the TruSeq stranded total RNA low-throughput (LT) sample prep kit for plants (Illumina, Inc., USA) from the total RNA. Sequencing was done by Macrogen, Inc. (Seoul, South Korea) using a HiSeq 2000 sequencer (Illumina, Inc., USA), generating a total of 60,085,298 reads. These sequences were trimmed and filtered using Trimmomatic 0.38. Duplicated sequences were removed using the dedupe tool on BBMap 38.34, and the remaining 10,841,600 reads between 50 and 151 bp long were assembled using SPAdes 3.13 with default parameters, generating a total of 185,467 contigs.

The assembled sequences were compared to other related sequences available in the GenBank Virus Reference database using BLASTn (5). One hundred forty-five contigs 78 to 2,263 bp long matched the LSV reference genome (NCBI RefSeq accession number NC_005138) (6), with a 95% identity threshold. These 145 contigs were further assembled using the Geneious 11.1.5 *de novo* assembler, with default parameters. The resulting final assembled sequence was 8,391 bp long (48.69% G+C content; *N*_50_ value, 1,268 bp) and had 97.1% identity with the reference LSV genome, which is 8,394 bp long, indicating that the complete genome was assembled. Alignment of the Ecuadorian LSV strain with other LSV complete genomes available in the NCBI database resulted in identities ranging from 97.1 to 84.7%; the most closely related genome has NCBI RefSeq accession number NC_005138 and was from South Korea, and the most distantly related genome has GenBank accession number LC004126 and was from Japan. Mapping deduplicated reads to the assembled LSV genome revealed a 1,451× sequencing depth of coverage. Analysis of the genome predicted six open reading frames (ORFs) on the positive strand, a 5′-untranslated region (UTR) of 55 nucleotides (nt), and a 3′-UTR of 48 nt, showing a typical organization of LSV, 5′-UTR-replicase-triple gene block-coat protein-6-kDa protein-3′-UTR. Alstroemeria mosaic virus was also found in the sample.

To our knowledge, this is the first announcement of a complete genome sequence of LSV in Alstroemeria plants in Ecuador.

### Data availability.

The complete genome sequence of LSV strain Ecuador has been deposited in the GenBank database under the accession number MK649770. Raw sequences were stored in the Sequence Read Archive under BioProject number PRJNA543330.
